# Supplementary material for: Splice-Junction-Based Mapping of Alternative Isoforms in the Human Proteome
Source: Cell Rep. Author manuscript; Available in PMC 2020 Jan 15. (PMC6961840; doi:10.1016/j.celrep.2019.11.026)
Supplement: 3 [file NIHMS1546469-supplement-3.zip › DF2/PXD006675/LeftVentricle-92-P52179-AAIAPPSPPCDITCLESFR.pdf]

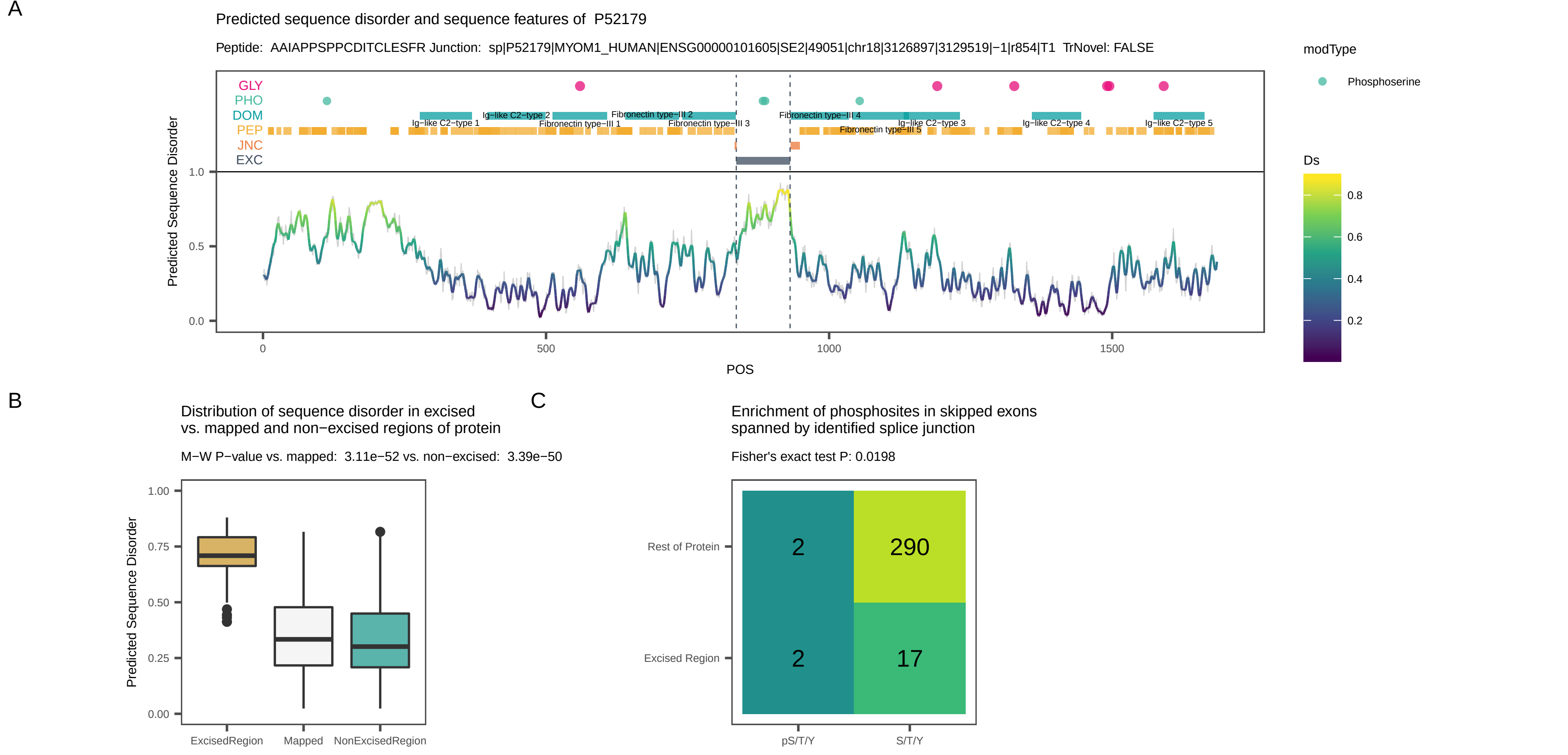
A
Predicted sequence disorder and sequence features of P52179
Peptide: AAIAPPSPPCDITCLESFR Junction: sp|P52179|MYOM1_HUMAN|ENSG00000101605|SE2|49051|chr18|3126897|3129519|−1|r854|T1 TrNovel: FALSE
GLY
PHO
DOM
PEP
JNC
EXC
Ig−like C2−type 1
Ig−like C2−type 2
Fibronectin type−III 1
Fibronectin type−III 2
Fibronectin type−III 3
Fibronectin type−III 4
Ig−like C2−type 3
Fibronectin type−III 5
Ig−like C2−type 4
Ig−like C2−type 5
Predicted Sequence Disorder
POS
modType
Phosphoserine
Ds
B
Distribution of sequence disorder in excised vs. mapped and non−excised regions of protein
M−W P−value vs. mapped: 3.11e−52 vs. non−excised: 3.39e−50
Predicted Sequence Disorder
ExcisedRegion
Mapped
NonExcisedRegion
C
Enrichment of phosphosites in skipped exons spanned by identified splice junction
Fisher's exact test P: 0.0198
Rest of Protein
Excised Region
pS/T/Y
S/T/Y
2
290
2
17
